# A case of acute myeloid leukemia (AML) with an unreported combination of chromosomal abnormalities: gain of isochromosome 5p, tetrasomy 8 and unbalanced translocation der(19)t(17;19)(q23;p13)

**DOI:** 10.1186/1755-8166-6-40

**Published:** 2013-09-30

**Authors:** Christian Paar, Gabriele Herber, Daniela Voskova, Michael Fridrik, Herbert Stekel, Jörg Berg

**Affiliations:** 1 Institute of Laboratory Medicine, General Hospital Linz, Krankenhausstrasse 9, A-4020, Linz, Austria; 2 Department of Medicine III, General Hospital Linz, Krankenhausstrasse 9, A-4020, Linz, Austria; 3 Institute of Laboratory Medicine, Medical University Graz, Auenbruggerplatz 2, A-8036, Graz, Austria

**Keywords:** AML, Acute monoblastic leukemia, Chromosomal abnormalities, Fluorescence in situ hybridization (FISH), Multicolor FISH, Tetrasomy 8, Isochromosome 5p, Clonal evolution

## Abstract

**Background:**

Acute myeloid leukemia (AML) comprises a spectrum of myeloid malignancies which are often associated with distinct chromosomal abnormalities, and the analysis of such abnormalities provides us with important information for disease classification, treatment selection and prognosis. Some chromosomal abnormalities albeit recurrent are rare such as tetrasomy 8 or isochromosome 5p. In addition, erratic chromosomal rearrangements may occur in AML, sometimes unbalanced and also accompanied by other abnormalities. Knowledge on the contribution of rare abnormalities to AML disease, progression and prognosis is limited.

Here we report a unique case of acute monoblastic leukemia with gain of i(5)(p10), tetrasomy 8, an unbalanced translocation der(19)t(17;19)(q23;p13.3) and mutated *NPM1*.

**Results:**

Bone marrow cells were examined by conventional karyotyping, fluorescence in situ hybridization (FISH) and mutation analysis at diagnosis and follow-up. At diagnosis we detected trisomy 8, an unbalanced translocation der(19)t(17;19)(q23;p13.3) and mutated *NPM1*. During the course of the disease we observed clonal evolution with gain of i(5)(p10), tetrasomy 8 and eventually duplication of der(19)t(17;19)(q23;p13.3). By using the der(19)t(17;19) as clonal marker, we found that i(5)(p10) and tetrasomy 8 were secondary genetic events and that tetrasomy 8 had clonally evolved from trisomy 8.

**Conclusions:**

This case of acute monoblastic leukemia presents a combination of rare chromosomal abnormalities including the unbalanced translocation der(19)t(17;19)(q23;p13.3), hitherto un-reported in AML. In addition, our case supports the hypothesis of a step-wise clonal evolution from trisomy 8 to tetrasomy 8 in AML. Reporting and collecting data of rare chromosomal abnormalities will add information to AML disease, progression and prognosis, and may eventually translate to improved patient management.

## Background

Acute myeloid leukemia (AML) comprises a spectrum of hematologic malignancies with variable outcomes. A hallmark of AML is maturation arrest of myeloid cells, which accumulate in the peripheral blood and bone marrow. The un-differentiated myeloid cells show chromosomal abnormalities in about 55% of cases of adult AML [1]. Some of these abnormalities, e.g. translocations are recurrent and are used for disease classification [1,2].

Cytogenetics has become mandatory for the diagnosis of AML according to the 2008 revision of the World Health Organization (WHO) classification of tumors of hematopoietic and lymphoid tissues [2]. Cytogenetic analyses complemented with molecular methods and mutation detection are useful for guiding treatment, for monitoring residual disease and for providing prognostic information towards clinical outcomes [3].

Trisomy 8 either as a sole or as an additional abnormality is the most common among numerical abnormalities associated with AML [4]. In contrast, tetrasomy 8 is relatively rare and reported in a few cases in AML, only [5-9]. Tetrasomy 8 seems to be associated with poor prognosis [5,8]. A clonal relationship between trisomy 8 and tetrasomy 8 has been suggested, however, hardly ever followed up in the cases described [8-10].

Gain of an isochromosome of the short arm of chromosome 5, i.e. i(5)(p10), represents a very rare recurrent abnormality in AML [11,12]. To date only few cases have been described (Table 1). The presence of i(5)(p10) seems to concomitantly occur with further abnormalities, sometimes in the context of complex karyotypes [13]. In most cases response to chemotherapy was reportedly poor [11-13].

**Table 1:** Cases of myeloid malignancies with presence or gain of i(5)(p10) described in the literature

**Case**	**FAB type**	**Karyotype**	**+i(5)(p10)**	**+8**	**References**
1	AML-M1	47,XX,t(1;19)(p22;q13),del(2)(q33),del(3)(q21),**+i(5)(p10)**,add(6)(p2?3)	+		Choi et al., 2007 [14]
2	AML-M1	48,XX,**+8**,+i(8)(q10)/49,idem,**+i(5)(p10)**	+	+	Calabrese et al., 1992 [15]
3	AML-M2^a^	46,XX,del(9)(q12q33)/46,idem,der(5)t(5;6)(q23;q22),der(6)t(5;6)(q35;q22),del(17)(p11)/46,idem,add(1) (p11),der(2)t(1;2)(p2?;p2?),der(2)t(2;12)(p2?;q14),del(5)(q14q34),i(5)(p10),add(12)(q2?),del(17)/46, idem,dic(4;5)(q11;q11),-5,**+8**,der(12)t(5;12)(q1?1;p1?)t(5;17)(q23;q11),-17,+mar/46,idem,dic(4;5),-5,**+8**, add(11)(p?),der(12)t(5;12)t(5;17),-17,-20,+r,+mar		+	Herry et al., 2007 [16]
					Herry et al., 2010 [13]
4	AML-M2	46,XY,i(5)(p10),+der(12)t(1;12)(p11;p13),-13,-17,?add(22)(q13),+der(?)t(?;13)(?;q12)/47,idem, del(1)(q11),+mar/47,idem,add(8)(q22),+mar			Tamura et al., 1995 [17]
5	AML-M3	47,XX,**+i(5)(p10)**,ins(15;17)(q22;q21q21)/48,idem,+9	+		Goldschmidt et al., 2010 [18]
6	AML-M4	47,XY,**+8**/47,XY,**+i(5)(p10)**/48,XY,**+i(5)(p10)**,**+8**	+	+	Panani, 2006 [12]
7	AML-M4	43-45,XY,add(2)(p?21),i(5)(p10),-7,**+8**,-12,-16,-17,add(17)(p11),+1-3mar,inc		+	El Rifai, 1997 [19]
8	AML-M5	46,XX,t(6;14)(p12;q32),t(8;16)(p11;p13)/45,idem,-10/47,idem,**+i(5)(p10)**	+		Schmidt et al., 2004 [20]
9	AML-M5	46,X,del(Y)(q12),+der(2)t(2;14)(p11;q11),**+i(5)(p10)**,**+8**,del(9)(q13),t(9;10)(q13;p11),del(10)(p11),-13,-14	+	+	Yunis et al., 1984 [21]
10	AML-M5a	47,XX,**+i(5)(p10)**,t(8;16)(p11;p13)	+		Gervais et al., 2008 [22]
11	AML-M5a	48,XX,**+i(5)(p10)**,**+8**	+	+	Schoch et al., 2001 [11]
12	AML-M5a	48,XX,**+i(5)(p10)**,**+8**	+	+	Schoch et al., 2001 [11]
13	AML-M5a	48,XX,**+i(5)(p10)**,**+8**,der(14)t(1;14)(q11;p11)	+	+	Schoch et al., 2001 [11]
14	AML-M5b	50,XY,+der(5)t(1;5)(p13;q11),**+i(5)(p10)**,add(6)(q25),**+8**,+der(8)t(8;14)(p11;q11),-14,del(17)(p11),+20	+	+	Slovak et al., 1991 [23]
15	AML-M6	46,XY,i(5)(p10),-10,add(12)(p?),-13,-14,-15,+4mar/46,XY,i(5),-6,-7,-17,-19, +rx3,+mar			Herry et al., 2010 [13]
16	s-AML^b^	48,XY,+1,der(1;13)(q10;q10),**+i(5)(p10)**,**+8**	+	+	Flach et al., 2011 [24]
17	MDS	46,XX,i(5)(p10)			Douet-Guilbert et al., 2011 [25]
18	MDS	46,XY,i(5)(p10),**+8**		+	Jimenez-Souza et al., 2010 [26]
19	RAEB-1	46-48,XX,i(5)(p10),+6,add(6)(p?)x2,-7,add(9),add(14)(p?),+mar			Lessard et al., 2007 [27]
20	RARS	47,XY,i(5)(p10),del(12)(p11),+i(12)(p10)			Christodoulou et al., 2004 [28]
21	RCMD	44,X,-Y,i(5)(p10),inc			Reddi et al., 2012 [29]
22	RCMD	46,XX,i(5)(p10),add(6)(p?),?i(9)(p?),add(14)(p10)/47,idem,+add(6)			Herry et al., 2010 [13]
23	CML	47,XX,+X,der(1)t(1;5)(q36;q11),t(3;9;22;12)(q12;q34;q11;p13),-5,i(5)(p10),**+8**		+	Markovic et al., 2000 [30]

s-AML, secondary acute myeloid leukemia; MDS, myelodysplastic syndrome; RAEB-1, refractory anemia with excess blasts-1; RARS, refractory anemia with ring sideroblasts; RCMD, refractory cytopenia with multilineage dysplasia.

Bold letters highlight gain of isochromosome 5p and/or trisomy 8.

^a^This case was reported by Herry et al. as patient 16 in 2007 [16] and as patient 5 in 2010 [13].

^b^This case was initially classified as RAEB-2.

Recurrent translocations in AML are typically balanced and are often associated with a favorable prognosis. Erratic chromosomal rearrangements may occur in AML, sometimes unbalanced and also accompanied by other abnormalities. They are thought to contribute to disease progression and are usually associated with poorer prognosis [3].

We present a unique case of AML with gain of i(5)(p10), tetrasomy 8, unbalanced translocation der(19)t(17;19)(q23;p13.3) and *NPM1* mutation. By following the course of the disease we show that gain of i(5)(p10) and tetrasomy 8 represent secondary events in this case and that tetrasomy 8 has clonally evolved from trisomy 8.

## Case presentation

In June 2011 a 64 year old male patient was admitted to our hospital with recurrent epistaxis and ecchymosis. The diagnosis of AML, in this case acute monoblastic leukemia, was established. As the patient was not eligible for bone marrow transplantation due to comorbidities, induction therapy (7+3) with daunorubicin and cytarabine followed by three cycles of consolidation treatment with high-dose cytarabine was administered and led to complete hematologic and cytogenetic remission. A first relapse occurred 14 months thereafter, which was treated with induction therapy as before and, again, resulted to complete remission. At 19 months a second relapse occurred with additional infiltration of inguinal lymph nodes and the skin. In January 2013 induction treatment with cisplatin, gemcitabine and dexamethasone was administered, which resulted in hematologic and partial cytogenetic remission at four month thereafter. In July 2013 the patient vastly relapsed and passed away despite of continued consolidation therapy.

## Results

**Figure 1:**
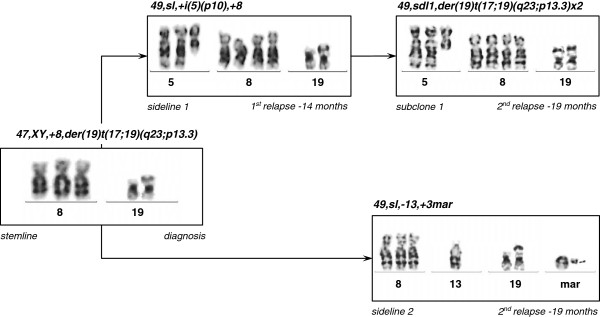
**Partial karyograms of clonal evolution.** GTG-banded partial karyograms of all the involved chromosomes show the step-wise clonal evolution from trisomy 8 to tetrasomy 8, gain of i(5)(p10) and derivative chromosome(s) 19 in the predominant sideline 1 at diagnosis, at first and second relapse (upper panel). The lower panel depicts sideline 2 with trisomy 8, monosomy 13, a derivative chromosome 19 and three marker chromosomes (second relapse). Note, no other aberrant clones have been detected at the respective time points.

**Figure 2:**
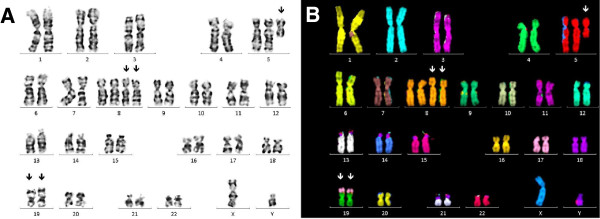
**GTG-banded karyotype and multicolor-FISH image at second relapse. (A)** The predominant aberrant subclone 1 shows gain of i(5)(p10), tetrasomy 8 and two derivative chromosomes 19. **(B)** Multicolor-FISH analysis confirmed the chromosomal aberrations and unveiled the unbalanced der(19)t(17;19). Arrows point to chromosomal abnormalities.

At diagnosis conventional cytogenetics revealed the following karyotype: 47,XY,+8,add(19)(p13)[5]/46,XY[15]. The initial karyotype was defined more precisely with multicolor fluorescence in situ hybridization (24X-FISH) analysis, which yielded to the following karyotype: 47,XY,+8,der(19)t(17;19)(q23;p13.3)[6]/46,XY[19]. At first relapse a marked clonal evolution had occurred with gain of isochromosome 5p and tetrasomy 8. The evolved karyotype was determined as: 49,XY,+i(5)(p10),+8,+8,der(19)t(17;19)(q23;p13.3)[7]/46,XY[14] (Figure 1). At second relapse cytogenetic analysis showed a duplication of the derivative chromosome 19 with loss of the normal chromosome 19 (Figures 1 and 2A). In addition, a second line was detected with trisomy 8, monosomy 13, der(19)t(17;19) and 3 marker chromosomes, however, no i(5)(p10).

**Figure 3:**
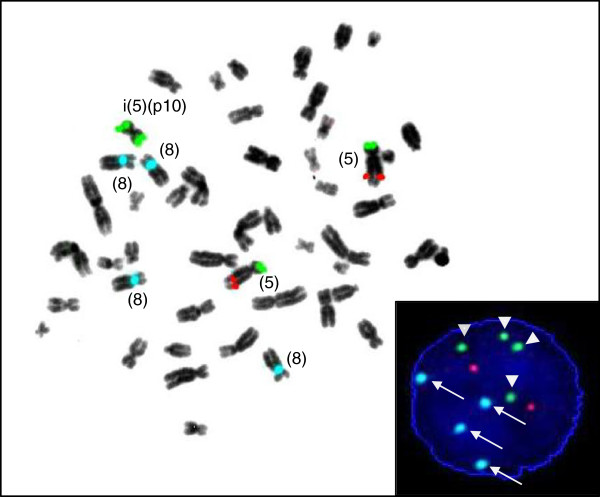
**FISH analysis using locus specific probes for chromosome 5p/5q and a centromer enumeration probe for the identification of chromosome 8.** Hybridization signals on an inverted DAPI counterstain metaphase spread show four blue signals indicative of tetrasomy 8, two green signals on the supernumerary isochromosome 5p and two green and two red signals on the normal chromosomes 5. The insert shows a representative interphase-FISH image of sideline 1/subclone 1 with the signal pattern of two red and four green dots (arrow-heads) indicating gain of i(5)(p10) and four blue dots indicating tetrasomy 8 (arrows).

**Figure 4:**
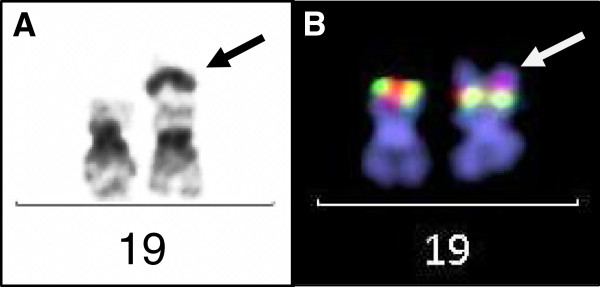
**Analysis of the derivative chromosome 19. (A)** Partial GTG-banded karyogram showing a normal and a derivative chromosome 19 (left- and right-hand side, respectively). The arrow points to the additive band from 17q23. **(B)** Metaphase-FISH analysis using the *E2A* gene specific break-apart probe (green, D19S883; red, RH98588), which shows two normal fusion signals on both chromosomes. The arrow points to the additive chromosomal material on the derivative chromosome 19, which is illuminated by the DAPI counterstain.

Triple-color metaphase- and interphase-FISH analysis with a probe mixture for chromosome 8 centromers and for chromosome 5p/5q complemented by 24X-FISH analysis confirmed the respective abnormalities (Figures 2B and 3). In order to narrow the breakpoints of der(19)t(17;19), metaphase-FISH with an *E2A* specific break-apart probe (D19S883) was used. Fusion signals were observed on both normal and derivative chromosomes 19, which indicated that the breakpoint on chromosome 19 ought to be located distal from the *E2A* gene locus (Figure 4). The final karyotype was determined as: 49,XY,+i(5)(p10),+8,+8,der(19)t(17;19)(q23;p13.3)x2[16]/49,XY,+8,-13,der(19)t(17;19)(q23;p13.3),+3mar[4]/46,XY[2] (Figure 1).

The mutation analysis for *NPM1* and *FLT3* revealed the *NPM1*-mutation c.959_960insCAGA and no *FLT3* internal tandem duplication, respectively. This was also obtained at relapses, and the *NPM1* mutation was conserved over the entire course of the disease.

At diagnosis the bone marrow aspirate showed 81% monoblasts which stained positive for HLA-DR, CD33, CD56, CD36, CD64, and negative for MPO, Tdt and CD117, and the CBC was as follows: WBC 26.7 G/L (47% blasts), Hgb 14.1 g/dl, and platelets 56 G/L. The immunophenotyping and the cytological analysis were consistent with acute monoblastic leukemia.

## Discussion

We described a case of acute monoblastic leukemia with a unique combination of rare aberrations, i.e. gain of i(5)(p10), tetrasomy 8, der(19)t(17;19)(q23;p13.3) and *NPM1* mutation. To the best of our knowledge such a case has not been reported previously.

In myeloid malignancies the occurrence of i(5)(p10) is very rare and to date only 23 cases have been reported in the literature (Table 1). The occurrence of i(5)(p10) was described in 16 cases of AML, in 6 cases of MDS and in one case of CML. Interestingly, gain of i(5)(p10), which results to a supernumerary chromosome 5, has exclusively been reported in only 12 cases of AML, however, not in MDS (Table 1). In 8 of the 12 cases with AML, i(5)(p10) was accompanied by trisomy 8. In 7 of the 12 cases the cells were of monoblastic/monocytic lineage (FAB M5). The gain of i(5)(p10) resulted to tetrasomy 5p and disomy 5q, which was also observed in our case. In contrast, in the cases with MDS (6 cases) and in other cases of AML (4 cases) an i(5)(p10) replaced a normal chromosome 5 and, thus, led to trisomy 5p and monosomy 5q, perhaps similar to a 5q- syndrome [13,25]. It is not clear, whether these differences translate into differing phenotypes of the diseases, as e.g. the cytological pattern is not completely consistent in the cases described (Table 1). With two exceptions, i(5)(p10) was always observed in the context of at least one other chromosomal abnormality, often in the context of a complex karyotype (Table 1). Thus, i(5)(p10) may represent a secondary cytogenetic event in the majority of cases [11,13]. Our case supports this notion, since gain of i(5)(p10) was not detected at diagnosis but was observed in the wake of clonal evolution.

In the past, the incidence of i(5)(p10) could have been underestimated by conventional G-banding as i(5)(p10) might have been misinterpreted as 5q-, which is frequent in patients with MDS or AML. By using locus specific probes towards 5p and 5q, FISH analysis allowed clear discrimination between 5q- and i(5)(p10) both on metaphases and on interphase nuclei (Figure 3). This approach is useful not only in this case but also in the routine diagnostic work-up of other cases.

Tetrasomy 8 has been reported in about 120 cases to date [5]. It was observed mostly in AML of monocytic/monoblastic lineage, less frequently in MDS and very rarely in MPD [5]. In the cases described the clone with tetrasomy 8 was often seen in the presence of trisomy 8, which favoured the hypothesis of a stepwise clonal evolution by consecutive mitotic non-disjunctions from disomy 8 to trisomy 8 and to tetrasomy 8 or polysomy 8 [5,6,8]. Alternatively, tetrasomy 8 may evolve from simultaneous non-disjunction of both homologous chromosomes during a single cell division [8]. In our case clonal evolution was monitored at three consecutive timepoints by using the derivative chromosome 19 as clonal marker. Thus, we demonstrated that tetrasomy 8 has evolved from trisomy 8. Clonal evolution of tetrasomy 8 in AML was previously reported in two cases, only. The one case by Kameoka et al. was a MDS with trisomy 8 as the sole abnormality, and when progression into AML occurred, tetrasomy 8 was found [9]. A chromosomal marker was not available as clonal control. In the other case by Takahashi et al. AML showed the translocation t(9;11)(p22;q23) and trisomy 8 at diagnosis [10]. During clonal evolution tetrasomy 8 was detected and the t(9;11)(p22;q23) served as clonal control. Together with our case these findings support the hypothesis that tetrasomy 8 usually evolves stepwise by consecutive non-disjunctions of chromosome 8 in AML [8].

Apart from trisomy 8 our case presented with an unbalanced translocation der(19)t(17;19)(q23;p13.3) at diagnosis. Although both breakpoint regions (17q23 and 19p13, respectively) are intermittently involved in various translocations and other aberrations in hematologic malignancies, a literature search (PubMed and Mitelman database) on t(17;19)(q23;p13) in AML did not return a single hit, which suggests that the observed derivative chromosome 19 ought be erratic in AML [31,32]. There is only one case of ALL described, which shows a balanced translocation t(17;19)(q23;p13) as sole abnormality [33]. Translocations, however, with a breakpoint at 19p13, often with involvement of *E2A,* have been reported in approximately 400 cases in AML and in the context of various fusion partners such as *MLL* on 11q23 and others [32]. In an approach to define the breakpoints on the molecular level in our case we used an *E2A* (*TCF3*) specific break-apart probe for chromosome-band 19p13 and obtained fusion signals only. This indicated absence of a chromosomal break within this gene locus (Figure 4). Therefore, we assume that the breakpoint is located distal from this locus (D19S883), if not within the sub-telomeric region. This also precludes other genes within this region with potential in leukemogenesis, such as *MLLT1* or *LYL1*, as those are located proximal to *E2A*. Distal to *E2A* are the genes *FSTL3* (*FLRG*), *PTBP1* and *STK11,* and there is only one report of involvement of *FSTL3* with *CCND1* in a case with B-CLL [34].

The band 17q23 has been identified as a “fragile site” in cancer with higher incidence of chromosomal breakage [35]. Nevertheless translocations involving 17q23 are rare in hematologic diseases and have been reported in AML in only 59 cases, of which 7 cases showed trisomy 8, too. Apart from protein coding genes in 17q23, there are four miRNA genes (*miR-21*, *miR-301*, *miR-142s*, *miR-142as*) located and involvement in neoplastic diseases has been suggested [36,37]. The translocation der(19)t(17;19)(q23;p13.3) in our case is new and further studies are warranted to unveil the potential involvement of genes in this unbalanced translocation. The underlying molecular event could have provided a proliferative advantage to the cells or assisted in genomic instability, since der(19)t(17;19)(q23;p13.3) was eventually found duplicated in the predominant clone.

AMLs with mutated *NPM1* harbour chromosomal abnormalities in only about 15% of cases and the most frequent abnormalities are +8, –4 or –Y [38,39]. As mutated *NPM1* is retained during clonal evolution, it is thought that *NPM1* mutations represent a founder mutation, and additional mutations or chromosomal abnormalities are thought to be secondary. In our case the *NPM1* mutation did not change during clonal evolution. However, as we observed trisomy 8 and der(19)t(17;19)(q23;p13.3) at diagnosis, it is not clear which of the abnormalities conferred the founder.

By using the International Prognostic Scoring System in AML our case initially classified as “Intermediate-II”, as trisomy 8 is not a favourable prognostic factor, but does not score as an adverse prognostic factor either [1,3]. However, the prognostic relevance of der(19)t(17;19)(q23;p13.3) is not known and, hence, this scoring was only provisional. At first relapse our case reclassified as “Adverse”, as the criteria of a complex karyotype were met with the addition of a fourth chromosome 8 and gain of i(5)(p10). Tetrasomy 8 has been described as a poor prognostic factor, however, much less information is available for i(5)(p10) [5,11,13]. On their own, tetrasomy 8 and i(5)(p10) are frequently part of a complex karyotype and may reflect genomic instability. Genes located on chromosome 8 and 5p may be over-expressed by gene dosage effects and, thus, could contribute to a possible proliferative advantage of the cells. *In vitro*, a proliferative advantage of tetrasomy 8 over trisomy 8 has been observed in AML [40]. Cases with either tetrasomy 8 or i(5)(p10) reportedly show a more aggressive course of the disease, shorter survival times and poor response to chemotherapy [11,13,25]. Those abnormalities are held as factors of poor prognosis and our case falls in line with those case reports.

## Conclusion

We described the first case of acute monoblastic leukemia with gain of i(5)(p10), tetrasomy 8, an unbalanced translocation der(19)t(17;19)(q23;p13.3) and *NPM1* mutation. During the follow-up of the disease we observed that gain of i(5)(p10) and tetrasomy 8 represented secondary genetic events in this case. By using the der(19)t(17;19) as clonal marker we formally demonstrated that tetrasomy 8 has evolved from trisomy 8. This case is only the third, illustrating a step-wise clonal evolution from trisomy 8 to tetrasomy 8 in AML.

Reporting and collecting data on rare chromosomal abnormalities of AML will add information to pathogenesis, disease progression and prognosis, and may eventually translate to improved patient management.

## Materials and methods

### Conventional karyotyping

Bone marrow aspirates were cultured for 24 h and 48 h in bone marrow culture medium (MarrowMax™, GIBCO, Paisley, UK). Metaphase chromosomes were prepared and GTG-banded by standard techniques. A minimum of 20 metaphases were karyotyped according to the International System for Human Cytogenetic Nomenclature (ISCN) 2013 [41].

### Fluorescence in situ hybridization (FISH)

Polysomy 8 and i(5)(p10) were simultaneously analyzed by using a probe mixture of the Vysis CEP 8 probe (D8Z2, SpectrumAqua) and the Vysis LSI 5p/5q Dual Color probe (EGR1/D5S23,D5S721, SpectrumOrange/SpectrumGreen, Abbott Molecular, Illinois, USA). The *E2A* (*TCF3*) gene locus on chromosome 19 was analyzed with the XL E2A break-apart probe (D19S883/RH98588, Metasystems, Altlußheim, Germany). Metaphase chromosomes were also analyzed with the 24XCyte multicolor FISH probe set (Metasystems). All hybridizations were performed on a ThermoBrite instrument (Abbott Molecular) with denaturation at 78°C for 2 minutes followed by over-night incubation at 37°C and subsequent standard washing procedures, which were completed by counterstaining with DAPI (4,6 diamidino-2-phenylindole). For interphase FISH cells from 24 h cultures were used, and more than 200 nuclei were analyzed. All of the FISH analyses were performed with a Zeiss Axio-Imager 1 M fluorescence microscope in combination with the Metafer 4 software (Metasystems).

### Mutation analysis

DNA and RNA were prepared from bone marrow aspirates by standard procedures and subjected to *NPM1* and *FLT3* gene mutation analysis as described [42,43].

### Morphology and Immunophenotyping

Peripheral blood cells were examined by an automated hematologic analyzer (Sysmex, XE-5000, Vienna, Austria). Bone marrow smears were stained with Wright-Giemsa and analyzed according to routine clinical laboratory procedures. Immunophenotyping of bone marrow cells was performed on an 8-color Navios™ Flow Cytometer (Beckman Coulter, Germany) according to the manufacturer’s instructions.

## Consent

The patient gave written informed consent for the publication of this case including accompanying images prior to his passing away. A copy of this statement is available for review by the Editor-in-Chief of this journal.

## Competing interests

The authors declared that they have no competing interests.

## Authors’ contributions

CP and GH performed the cytogenetic studies, DV and MF collected and compiled clinical data, JB and CP designed the study, HS participated in the design of the study and its coordination, JB and CP drafted the manuscript and all authors read and approved the final manuscript.
